# Comprehensive visualization of cell–cell interactions in single-cell and spatial transcriptomics with NICHES

**DOI:** 10.1093/bioinformatics/btac775

**Published:** 2022-12-02

**Authors:** Micha Sam Brickman Raredon, Junchen Yang, Neeharika Kothapalli, Wesley Lewis, Naftali Kaminski, Laura E Niklason, Yuval Kluger

**Affiliations:** Department of Anesthesiology, Yale School of Medicine, New Haven, CT 06511, USA; Department of Biomedical Engineering, Yale University, New Haven, CT 06511, USA; Pulmonary, Critical Care, and Sleep Medicine, Yale School of Medicine, New Haven, CT 06511, USA; Department of Immunobiology, Yale University, New Haven, CT 06511, USA; Interdepartmental Program in Computational Biology and Bioinformatics, Yale University, New Haven, CT 06511, USA; Pulmonary, Critical Care, and Sleep Medicine, Yale School of Medicine, New Haven, CT 06511, USA; Interdepartmental Program in Computational Biology and Bioinformatics, Yale University, New Haven, CT 06511, USA; Pulmonary, Critical Care, and Sleep Medicine, Yale School of Medicine, New Haven, CT 06511, USA; Department of Anesthesiology, Yale School of Medicine, New Haven, CT 06511, USA; Department of Biomedical Engineering, Yale University, New Haven, CT 06511, USA; Interdepartmental Program in Computational Biology and Bioinformatics, Yale University, New Haven, CT 06511, USA; Department of Pathology, Yale School of Medicine, New Haven, CT 06511, USA; Applied Mathematics Program, Yale University, New Haven, CT 06511, USA

## Abstract

**Motivation:**

Recent years have seen the release of several toolsets that reveal cell–cell interactions from single-cell data. However, all existing approaches leverage mean celltype gene expression values, and do not preserve the single-cell fidelity of the original data. Here, we present *NICHES* (*N*iche *I*nteractions and *C*ommunication *H*eterogeneity in *E*xtracellular *S*ignaling), a tool to explore extracellular signaling at the truly single-cell level.

**Results:**

NICHES allows embedding of ligand–receptor signal proxies to visualize heterogeneous signaling archetypes within cell clusters, between cell clusters and across experimental conditions. When applied to spatial transcriptomic data, NICHES can be used to reflect local cellular microenvironment. NICHES can operate with any list of ligand–receptor signaling mechanisms, is compatible with existing single-cell packages, and allows rapid, flexible analysis of cell–cell signaling at single-cell resolution.

**Availability and implementation:**

NICHES is an open-source software implemented in R under academic free license v3.0 and it is available at http://github.com/msraredon/NICHES. Use-case vignettes are available at https://msraredon.github.io/NICHES/.

**Supplementary information:**

Supplementary data are available at *Bioinformatics* online.

## 1 Introduction

Cellular phenotype across tissues and organs is heavily influenced by the biological microenvironment in which a given cell resides (Baccin *et al.*, 2020; Davidson *et al.*, 2020; McCarthy *et al.*, 2020; Nabhan *et al.*, 2018; Qadir *et al.*, 2020; Rodda *et al.*, 2018; Tikhonova *et al.*, 2020; Zhou *et al.*, 2018). Understanding the influence of cell–cell signaling on cell phenotype is a major goal in developmental and tissue biology and has profound implications for our ability to engineer tissues and next-generation cellular therapeutics. Single-cell technologies, which capture information both from individual cells and their surrounding cellular environment at the same time, are uniquely suited to exploring phenotype–environment relations. Many techniques are available to extract and prioritize extracellular signaling patterns from single-cell data, reviewed well in Dimitrov *et al.* (2022) and Armingol *et al.* (2021), including CellPhoneDB (Efremova *et al.*, 2020), NicheNet (Browaeys *et al.*, 2019), CellChat (Jin *et al.*, 2021), Connectome (Raredon *et al.*, 2022), SingleCellSignalR (Cabello-Aguilar *et al.*, 2020), iCELLNET (Noël *et al.*, 2021), Cellinker (Zhang *et al.*, 2021b), CellCall (Zhang *et al.*, 2021a) and PyMINEr (Tyler *et al.*, 2019). All of these techniques, however, rely on mean expression values calculated from single-cell clusters. Mean expression representation does not take full advantage of the single-cell resolution of the original measurements, thereby obscuring the rich repertoire of signaling patterns between cells. The field can benefit from a tool to assesses cell–cell signaling at the truly single-cell level, so that intra- and inter-cluster signaling patterns can be explored within observed data.

Here, we describe NICHES (Niche Interactions and Communication Heterogeneity in Extracellular Signaling), a software package to characterize cellular interactions in ligand–receptor signal-space at the single-cell level and to allow cross-platform low-dimensional embeddings of the resulting information. NICHES is designed for analysis of two types of cellular interactions: cell–cell signaling (defined as the signals passed between cells, determined by the ligand expression of the sending cell and the receptor expression of the receiving cell) and cellular niche (defined as the signaling input to a cell, determined by the ligand expression of surrounding or associated cells and the receptor expression of the receiving cell). The outputs from NICHES may be analyzed using existing single-cell software including Seurat (Butler *et al.*, 2018), Scanpy (Wolf *et al.*, 2018), Scater (McCarthy *et al.*, 2017) and Monocle3 (Cao *et al.*, 2019), thereby allowing deep computational analysis of cell signaling systems topology unapproachable with existing tools.

## 2 Approach

**Fig. 1.**
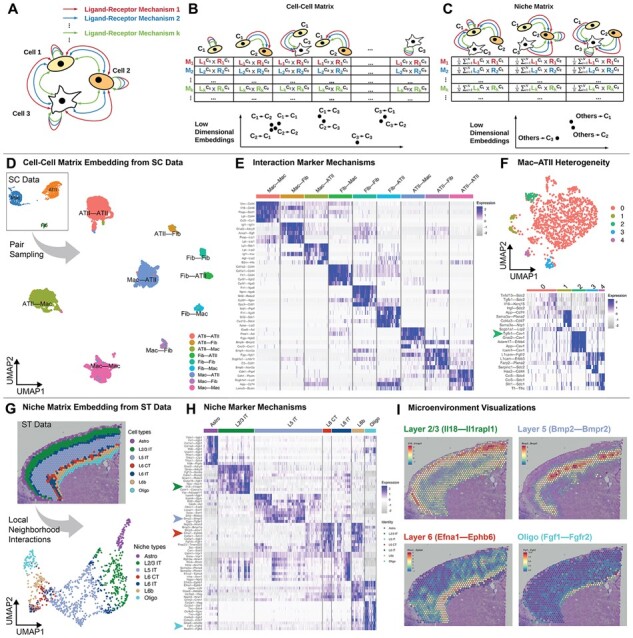
NICHES allows analysis of cell–cell interactions with single-cell resolution. (**A**) A set of cells may interact through many different ligand–receptor mechanisms. (**B**) NICHES represents cell–cell interactions as columns whose entries are calculated by multiplying ligand expression on the sending cell with receptor expression on the receiving cell, for each signaling mechanism. Low-dimensional embeddings may then be made of cell–cell interactions. Note schematic clustering of similar profiles. (**C**) Cellular microenvironments, or niches, of each cell are represented as columns calculated by multiplying mean ligand expression in the system with receptor expression on the receiving cell. This allows low-dimensional embedding of a proxy for sensed microenvironment for each cell. (**D**) NICHES analysis of single-cell (SC) data of three cell types co-localized in the rat pulmonary alveolus yields a quantitative cell–cell signaling atlas visualized by low-dimensional embedding. (**E**) Biologically relevant marker mechanisms may be identified for each celltype–celltype interaction. Because single-cell fidelity is preserved, NICHES allows observation of fine intra-cluster heterogeneity unobservable using mean-wise techniques. (**F**) Further analysis of a single celltype–celltype cross allows identification of mechanisms marking only subsets of cell pairings (see Tgfb1-Cav1 in this instance, arrow, which marks Cluster 2). (**G**) Local microenvironment may be estimated from spatial transcriptomic (ST) datasets by limiting cell–cell interactions to those within local neighborhoods, yielding a ‘niche’ atlas for each transcriptomic spot, which may be visualized in low dimensional space. (**H**) Signaling mechanisms marking the microenvironments of selected celltypes. Fgf1-Fgfr2 (bottom arrow) is a known potent regulator of oligodendrocyte phenotype (Furusho *et al.*, 2015; 2020) and here is found to be associated with oligodendrocyte-labeled spots. (**I**) Microenvironment mechanisms may be directly visualized *in situ*

NICHES takes single-cell data as input and constructs matrices where the rows are extracellular ligand–receptor signaling mechanisms and the columns are cell–cell extracellular signaling interactions (Fig. 1A–C, Supplementary Fig. S1). Cell–cell interactions are represented as columns whose entries are created by multiplying ligand expression on the sending cell with receptor expression on the receiving cell, for each mechanism (Fig. 1B, see Supplementary Software Methods). Cellular niches, an estimate of cellular microenvironment, are represented as columns that are created by multiplying mean ligand expression from sets of sending cells with the receptor expression on receiving cells (Fig. 1C, see Supplementary Software Methods). Row names are defined by the ground-truth ligand–receptor mechanism list set by the user. NICHES provides built-in access to ligand–receptor lists from the OmniPath and FANTOM5 databases (Ramilowski *et al.*, 2015; Türei *et al.*, 2021) and is compatible with custom mechanism lists containing any number of ligand or receptor subunits (Supplementary Fig. S2, see Supplementary Software Methods).

When applied to spatial transcriptomic data, interactions may be constrained by users to those occurring between spatial neighbors. The NICHES workflow, in this case, goes beyond colocalization-focused approaches developed for imaging mass cytometry (Schapiro *et al.*, 2017; Somarakis *et al.*, 2019; Stoltzfus *et al.*, 2020) and explicitly characterizes ligand–receptor connectivity within local neighborhoods. When applied to single-cell data, NICHES assumes full cellular connectivity for niche interactions and samples unique cell pairs from each celltype–celltype cross for cell–cell interactions. Biological assumptions, limitations to the mathematical formalism, best practice recommendations and detailed methods are provided in the Supplementary Material. Replicable vignettes covering a wide range of biological use-cases are available both within the NICHES software package and at https://msraredon.github.io/NICHES/.

## 3 Application

### 3.1 Advantages of NICHES over existing techniques

Because NICHES does not leverage cluster-wise mean values, signaling heterogeneity hidden by existing cell–cell signaling inference tools is easily observed (Supplementary Fig. S3, Supplementary Text). Archetype shifts between conditions with conserved mean expression may also be observed, which are difficult to capture with existing tools (Supplementary Fig. S4, Supplementary Text). NICHES also uniquely allows users to explore changes in system-level signaling due to the addition or loss of cell populations, a task which is not possible with current methods (Supplementary Fig. S5, Supplementary Text).

### 3.2 Cell–cell signaling atlases

NICHES allows comprehensive visualization of ligand–receptor patterns that are present in single-cell systems data (Fig. 1D–F). A uniform sample is taken of every celltype–celltype interaction resulting in a cell–cell signaling atlas that can be viewed via low-dimensional embedding (Fig. 1D). Celltype–celltype interactions generally display quantifiable signaling signatures as well as intra-relationship heterogeneity (Fig. 1E). Individual celltype–celltype crosses may be subclustered to further explore relationship heterogeneity and to identify mechanisms marking subtypes of cell–cell crosses (Fig. 1F).

### 3.3 Mapping local microenvironment in spatial atlases

NICHES can estimate local microenvironment in spatial transcriptomic data. Interactions may be limited to spatial nearest neighbors, allowing an estimation of local niche for each transcriptomic spot (Fig. 1G). Celltypes generally display stereotyped niche signatures with observable intra-niche heterogeneity (Fig. 1H). NICHES can reveal tightly localized microenvironments and mechanisms which can be visualized in spatial context (Fig. 1I). Sub-clustering can reveal microenvironment heterogeneity associated with tissue boundaries and transition regions (Supplementary Fig. S6).

### 3.4 Differential analysis across conditions and archetypes

NICHES allows flexible differential analysis of cell-to-cell signaling, system-to-cell signaling and cell-to-system signaling in both spatial and single-cell datasets. For brevity, we have compiled a series of vignettes online (https://msraredon.github.io/NICHES/) demonstrating specific use-cases. Best practices are discussed in the Supplementary Material.

## 4 Conclusion

NICHES is a flexible and powerful tool to explore cell–cell signaling interactions in single-cell and spatial transcriptomic data. NICHES supplements the capabilities of current techniques, allows single-cell resolution of niche signaling and cell–cell interactions, and establishes rich representations to analyze environment–phenotype relationships in tissues.

## Supplementary Material

btac775_Supplementary_Data

## Data Availability

The data underlying this article are available in the article and in its online supplementary material. Data used in vignettes may also be accessed through Zenodo, at https://doi.org/10.5281/zenodo.6846813, https://doi.org/10.5281/zenodo.6846617, https://doi.org/10.5281/zenodo.6878944, and https://doi.org/10.5281/zenodo.6878953.
