# Supplementary material for: Comprehensive visualization of cell–cell interactions in single-cell and spatial transcriptomics with NICHES
Source: Bioinformatics. 2022 Dec 2;39(1):btac775. doi: 10.1093/bioinformatics/btac775 (PMC9825783; doi:10.1093/bioinformatics/btac775)
Supplement: btac775_Supplementary_Data [file btac775_supplementary_data.pdf]

Supplementary Materials for

Comprehensive visualization of cell-cell interactions in single-cell and spatial transcriptomics with NICHES

Authors
Micha Sam Brickman Raredon[1,2,3,4,†,*] and Junchen Yang[5,†], Neeharika Kothapalli[3], Wesley Lewis[5], Naftali Kaminski[3], Laura E. Niklason[1,2], Yuval Kluger[5,6,7,*]

Contact: michasam.raredon@yale.edu; yuval.kluger@yale.edu

This PDF files includes:
Supplemental Text
Supplemental Findings
Supplemental Figures
Software Methods
Application Methods
Supplemental References

Software and Vignettes associated with this manuscript are available here:
https://msraredon.github.io/NICHES/
https://github.com/msraredon/NICHES
10.5281/zenodo.6846813

Data used in Vignettes is available at:
10.5281/zenodo.6846617
10.5281/zenodo.6878944
10.5281/zenodo.6878953

Supplemental Text

Workflow

The main function of our method is *RunNICHES* (Figure S1). It takes a single-cell or spatial gene expression matrix and the associated cell metadata as input and the external ligand-receptor mechanism databases as reference, and builds different cell interaction matrices (e.g. the Cell-Cell Matrix and NICHES Matrix) as specified by the user. It further aggregates and assembles the user-specified metadata accordingly. The exact matrix computation is described in detail in the Supplementary section *Cell-Cell Matrix Construction* and *Niche Matrix Construction*.

For the output, users can easily convert the cell interaction matrices and other information into formats for different single cell data analysis frameworks as they prefer, as demonstrated in our online vignette (https://msraredon.github.io/NICHES/articles/08%20Interoperability%20-%20NICHES.html), and then perform downstream analysis.

NICHES generates all user-requested cell-interaction matrices along with associated compiled metadata required for downstream data visualization, clustering, and differential analysis. The outputs of NICHES include:

- *Assay:* Stores the cell interaction matrix and associated data transformations.
- *Metadata:* Stores the aggregated metadata information, as mentioned in the Supplemental Methods, for every input metadata category (e.g., cell types, sample, batch). The aggregated metadata will include the sending metadata, receiving metadata, and sending-receiving metadata as appropriate for a given output. We argue that by carrying over the metadata (i.e., sample labels, coarse- and fine-grain cluster labeling, experimental conditions) from source data, NICHES allows rapid downstream differential analysis between already tagged groupings of cells.

Further associated data frameworks may then be generated including:

- *Reductions:* Stores the dimensional reductions for data visualization purposes (e.g., PCA, UMAP, TSNE).
- *Graphs:* Stores computed nearest neighbor graphs to perform graph-based clustering algorithms (e.g., Louvain clustering). Corresponding cluster labels will be stored in the *Metadata* table. Clustering will help to discover the heterogeneity of cell interaction signals in the data.

Assumptions, Limitations, and Best Practices

1) NICHES is an extracellular connectivity tool designed to quantify the likelihood of cell-cell communication based on cognate ligand-receptor expression between cells. Complete biological communication requires not only this cognate ligand-receptor expression but also appropriate cellular co-localization for a given mechanism, evidence of signal transduction,

absence of downstream signal inhibition, and subsequent phenotypic response, which is cell-state dependent. We think of NICHES as quantifying the mechanistic ‘wavelengths’ that a sending and receiving cell are jointly ‘tuned’ to.

2) In its present form, NICHES multiplies ligand expression on sending cells with receptor expression on receiving cells. This operator was chosen because it is zero-preserving. We would like to note that simple multiplication of ligand and receptor expression values does not necessarily reflect the modulation of phenotypic responses by involved cells. Such effects are often non-linear and do not necessarily correlate with ligand and receptor expression. However, multiplying sending and receiving values does provide a reasonable big-picture proxy of cellular connectivity within a dataset that allows organization of the data, similarly to practices in graph-network-based approaches that are widely used in genomics. Multiplication of ligand and receptor information, either via product or geometric mean, is used as part of the workflows in CellChat (Jin, et al., 2021), iCellNet (Noël, et al., 2021), NATMI (Hou, et al., 2020), SingleCellSignalR (Cabello-Aguilar, et al., 2020), SoptSC (Wang, et al., 2019), CellCall (Zhang, et al., 2021), and CommPath (Lu, et al., 2022), among others (Almet, et al., 2021).

3) When mechanisms are queried which containing more than one ligand subunit or more than one receptor subunit, NICHES multiplies the expression of all ligand subunits to yield a single ligand-mechanism expression value and multiplies the expression of all receptor subunits to yield a single receptor-mechanism expression value (see Figure S1 for a diagram of this computation). We have chosen this approach so that zero expression of any subunit within a given mechanism yields zero connectivity for that mechanism. It should be noted, however, that the sparse nature of single-cell transcriptomic data means that NICHES may output zero connectivity for a given mechanism between two cells if all subunits were not captured in a cell used for a given cell-cell pairing. Imputation prior to running NICHES can lessen this artifact, at the cost of using pseudo-values to calculate connectivity. For a demonstration of this difference, please see https://msraredon.github.io/NICHES/articles/02%20NICHES%20Single.html

4) The SystemToCell and CellToSystem outputs estimate connectivity between an entire single-cell system and each individual cell within that system. These tools are designed to quantify changes in system signaling character due either to altered cellular gene expression or to altered cellular distribution or representation. These tools use the mean operator to combine ligand information from multiple sending cells and/or to combine receptor information from multiple receiving cells.

5) When spatial information is provided, NICHES allows users to limit analysis to connectivity within either a defined radius or to local nearest-neighbor communities. However, when spatial information is not provided, NICHES treats all barcodes within an input system as available for sampling and analysis. If cells have been captured which are not to be treated as within a biological system of interest, we recommend removing these cells from the input data prior to running the SystemToCell or CellToSystem functionality, as otherwise ligand or receptor information from these cells will be included downstream.

6) We recommend standardizing total input cell number to maximize the accuracy of cross-system system-level comparisons. We make this recommendation because artifacts may otherwise theoretically be created in instances in which variable cell-number denominators act on constant system-expression numerators for a given mechanism.

7) Batch effects which cause biologically artifactual variance in ligand and receptor expression will affect the outputs from NICHES. This should be noted by users.

8) Differential testing may be performed on NICHES output data. If comparing experimental conditions containing multiple batches, users may run NICHES either for each batch independently or for an entire condition (containing multiple batches) at once. Running NICHES on individual batches will preserve batch effects for downstream analysis. Conversely, running NICHES on an experimental condition containing multiple batches will have the effect of smoothing cell-signaling batch effects, since cells will be paired with other cells within their condition but not necessarily within their specific batch.

9) If a single dataset is input into NICHES which contains information across multiple experimental conditions, cells may be paired with cells outside of their respective condition. We currently do not recommend doing this unless such patterns are deliberately of interest to a user.

Supplemental Findings

Simulation 1: NICHES reveals hidden intra-cluster signaling heterogeneity

To demonstrate NICHES's ability to discover intra-cluster heterogeneity, we generated a synthetic single-cell RNA-seq dataset with 400 cells equally divided into 2 cell types (C1 and C2), which can be separated by their marker genes (Figure S3, A, top).

Additionally, there are 2 subtypes S1 and S2 within C1 and 2 subtypes S3 and S4 within C2 (Figure S3, A, bottom). By design, S1 and S2 interact differentially with S3 and S4 through 2 ligand-receptor mechanisms. Specifically, S2 has a higher expression of *A2m* ligand gene compared to S1 (corresponding receptor gene is *Lrp1* that is highly expressed in S3 and S4), and S3 has a higher expression of receptor gene *Agtr2* compared to S4 (corresponding ligand gene is *Ace* which is highly expressed in S1 and S2), as shown in Figure S3, B. Due to the subtle gene expression difference, one cannot distinguish the subtypes based on the overall gene expression profiles. But because the nuance involves ligand and receptor genes, we may apply NICHES to capture this signal.

Figure S3, C, shows the cell-cell matrix output of NICHES, embedded in 2D mechanism space, clustered via k-means (top) and labeled by ground-truth subtype interaction (bottom). Sending cells are all from C1 and receiving cells are all from C2, and the space is 2-dimensional because there are only 2 ligand-receptor mechanisms in this simulation: *A2m-Lrp1* and *Agtr2-Ace*. Note that the clustering results (Figure S3, C, top) are almost completely identical to the ground truth subtype interaction pair labels (Figure S3 C, bottom). For reference, we have added a single black point representing the single mean connectivity value between C1 and C2 which results from using a mean-wise computational method to assess ligand-receptor connectivity in this simulation.

Simulation 2: NICHES preserves information regarding differential signaling distributions in disparate experimental conditions

Next, we sought to demonstrate that NICHES can register how cellular archetype shift influences cell-cell connectivity. We simulated 2 scRNAseq cases, both of which contain 2 cell types (C1 and C2), as shown in Figure S4, A. In both cases, we simulated 1 active ligand-receptor channel between C1 and C2 (C1 expresses the ligand gene A2m and C2 expresses the receptor gene Lrp1). C1 expression level of ligand A2m is sparse but high in Case 1, and broad but low in Case 2, with similar mean values (Figure S4, B). By design, the expression level of receptor by population C2 is identical in both cases. Because the mean expression level of A2m is similar, the mean connectivity of A2m-Lrp1 is similar between the 2 cases (Figure S4, C). NICHES, however, is able to detect this significant difference in connectivity (Figure S4, D).

Simulation 3: NICHES allows high-dimensional visualization of altered systems-cell topology due to addition or removal of cells

Next, we sought to evaluate the ability of NICHES to map changes in system-to-cell signaling topology. We simulated two cell systems to be compared. The first contains two cell types (C1 and C2, Figure S5,

A) while the second contains 3 cell types (C1, C2, and C3, Figure S5 B). C1 and C2 are identical in character in each case, and are connected via two simulated mechanisms, A2m-Lrp1 and Ace-Agtr2, where C1 cells express the ligands A2m and Ace and C2 cells express the receptors Lrp1 and Agtr2. C3, which is only present in the three-cell system, also expresses ligands A2m and Ace. From the perspective of a receiving cell (from population C2, in this simulation), the mean expression of ligand is higher in the three-cell system as compared to the two-cell system. NICHES quantifies this difference and allows analysis and visualization between conditions (Figure S5, C).

Supplemental Figures

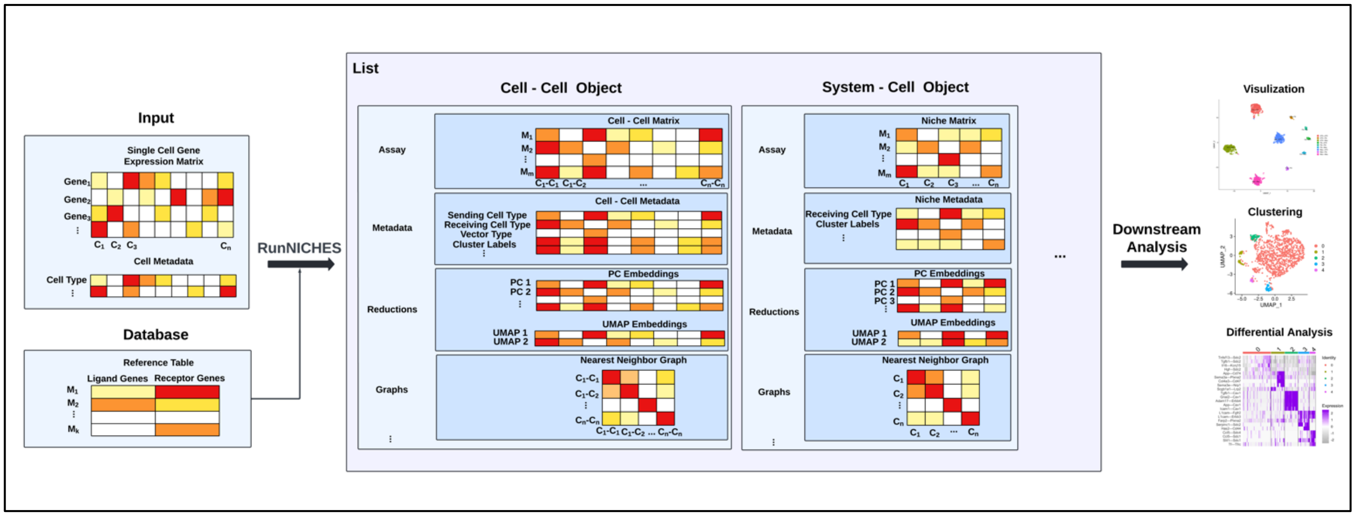

Figure S1: NICHES data infrastructure and workflow

NICHES takes a single-cell or spatial gene expression matrix (columns are cells, rows are genes) and the associated cell metadata (e.g., cell types, sample IDs, batch information) as input. By referencing the ligand-receptor interaction mechanisms via external databases (e.g., Fantom 5, OmniPath, or other user-customized databases), NICHES builds cell interaction matrices (e.g., Cell – Cell Matrix where columns are cell pairs and rows are interaction mechanisms, and Niche Matrix where columns are niche environments and rows are interaction mechanisms) via *RunNICHES* function. These new cell-signaling objects may be output as either Seurat objects from the Seurat package or as tabular matrices that are cross-compatible with multiple single-cell platforms. Here, we schematize the outputs from NICHES and additional data forms which may be computed from these outputs. Cell interaction matrices are output (Assay) along with other useful information (e.g., aggregated Metadata) and may be processed to yield data embeddings (Reductions) and nearest neighbor graphs (Graphs) for downstream analysis (e.g., data visualization, clustering, and differential analysis).

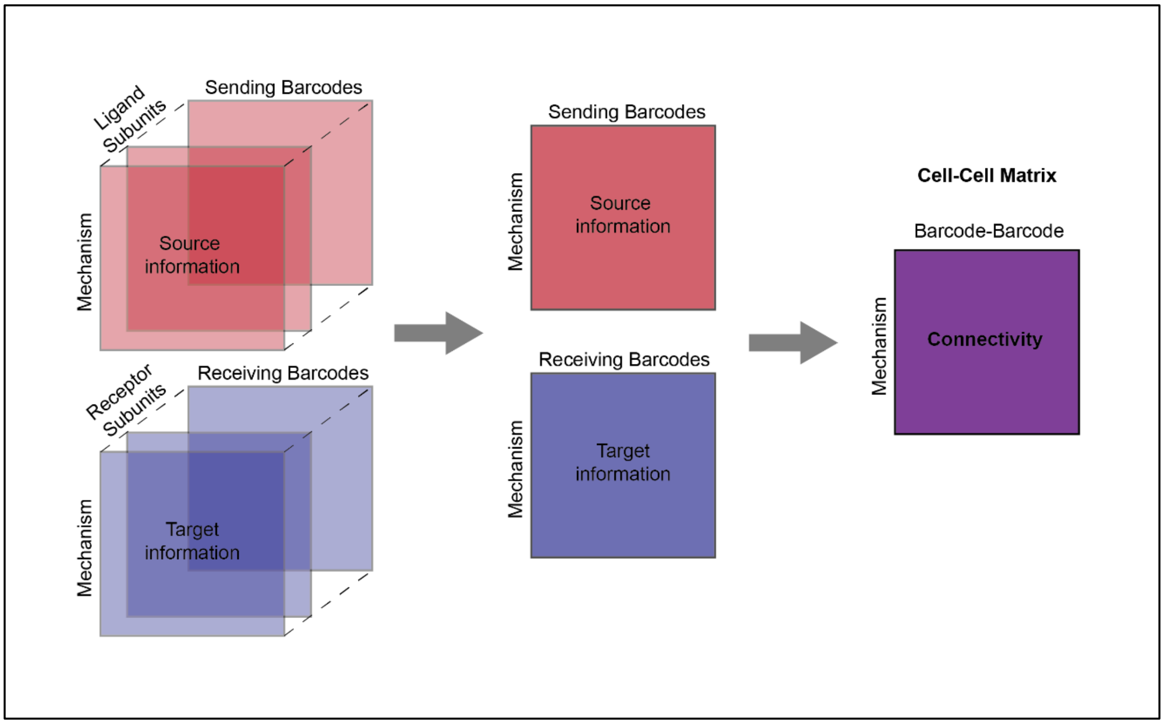

Figure S2: NICHES connectivity for mechanisms with multiple subunits

NICHES is capable of calculating intercellular connectivity for ligand-receptor mechanisms with any number of subunits. For a given mechanism, ligand subunit expressivities on the sending cell are multiplied together and receptor subunit expressivities on the receiving cell are multiplied together. These two values are then multiplied to yield mechanism connectivity between the sending and receiving cell. This operation is zero-preserving by design, so that the lack of expression of even a single subunit on either the sending or receiving cell within a given cell-cell pairing will cause a connectivity value of zero.

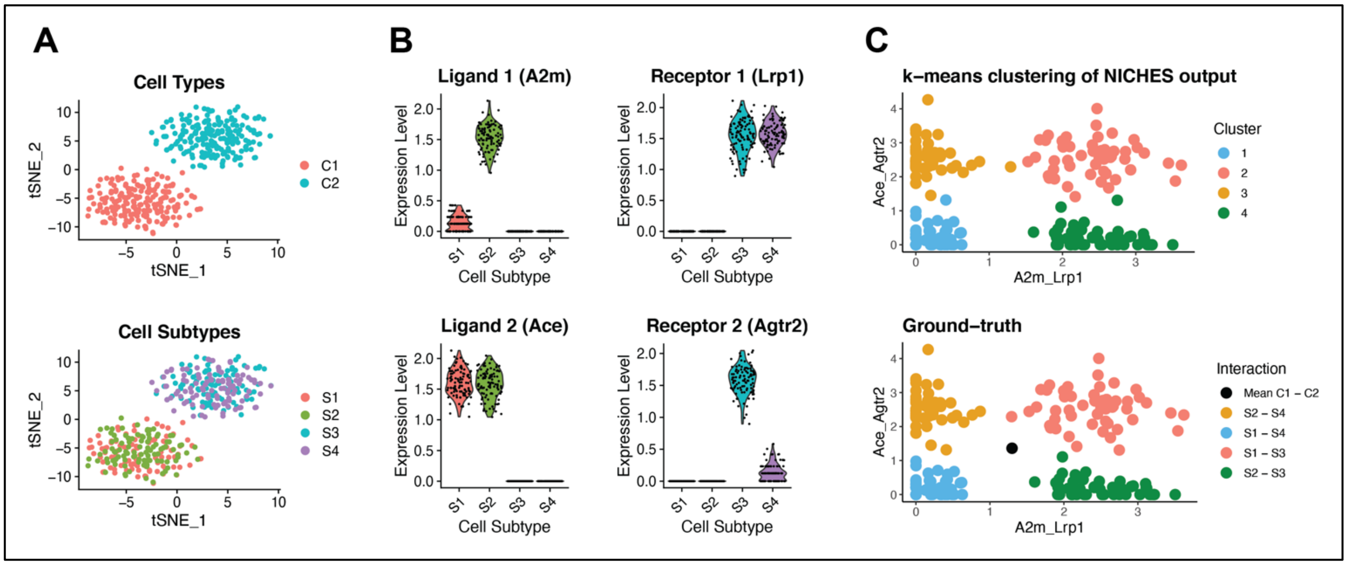

Figure S3: NICHES captures heterogeneity in cell-cell connectivity

In this simulation using synthetic data (see Methods) there are two celltypes labeled C1 and C2 containing signaling subtypes S1-S4 which do not resolve in gene space (A). These subtypes communicate in distinct ways via two distinct signaling mechanisms: A2m-Lrp1 and Ace-Agtr2 (B). Subpopulation S2 expresses ligand A2m higher than S1 while subpopulation S3 expresses receptor Agtr2 higher than S4. This expression pattern creates four distinct cell-cell signaling relationships even though only two celltypes have been considered. NICHES allows rapid observation of these distinct relationships using two-dimensional embeddings and k-means clustering (C, top) which closely matches the ground truth subtype pairings in this simulation (C, bottom). Mean connectivity between C1 and C2 is represented in black in the lower panel of (C). A biological counterpart to this simulation is show in Figure 1F.

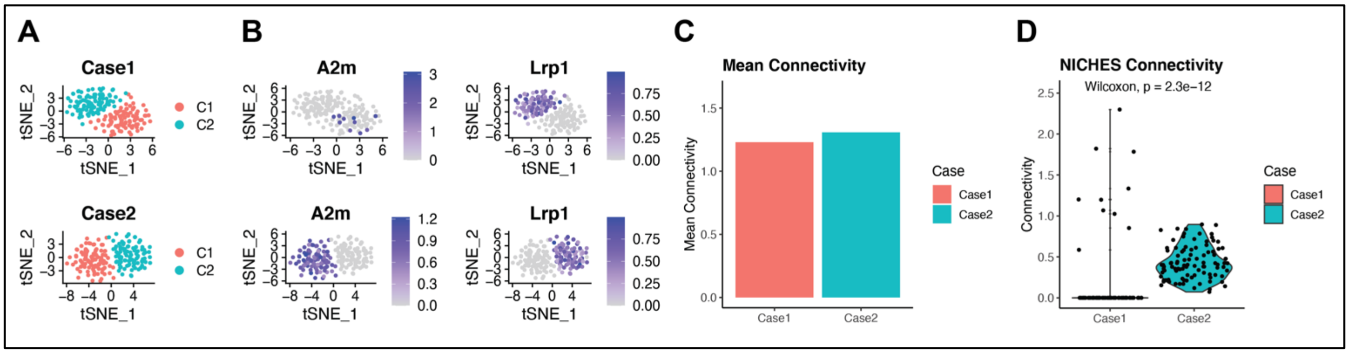

Figure S4: NICHES captures cross-condition archetype shift in cell-cell connectivity

In this simulation using synthetic data (see Methods), we compare two cell-systems (A, Case 1 and Case 2) representing different experimental conditions or tissues containing the same number of cells, with the same celltypes present, and similar mean connectivity for a given signaling mechanism. Case 1 cells express ligand sparsely but highly, while Case 2 sending cells express ligand broadly but lowly (B). Receptor expression is identical in each case. While mean connectivity is nearly identical (C), NICHES captures the significantly different ground-truth connectivity between the two cases (D).

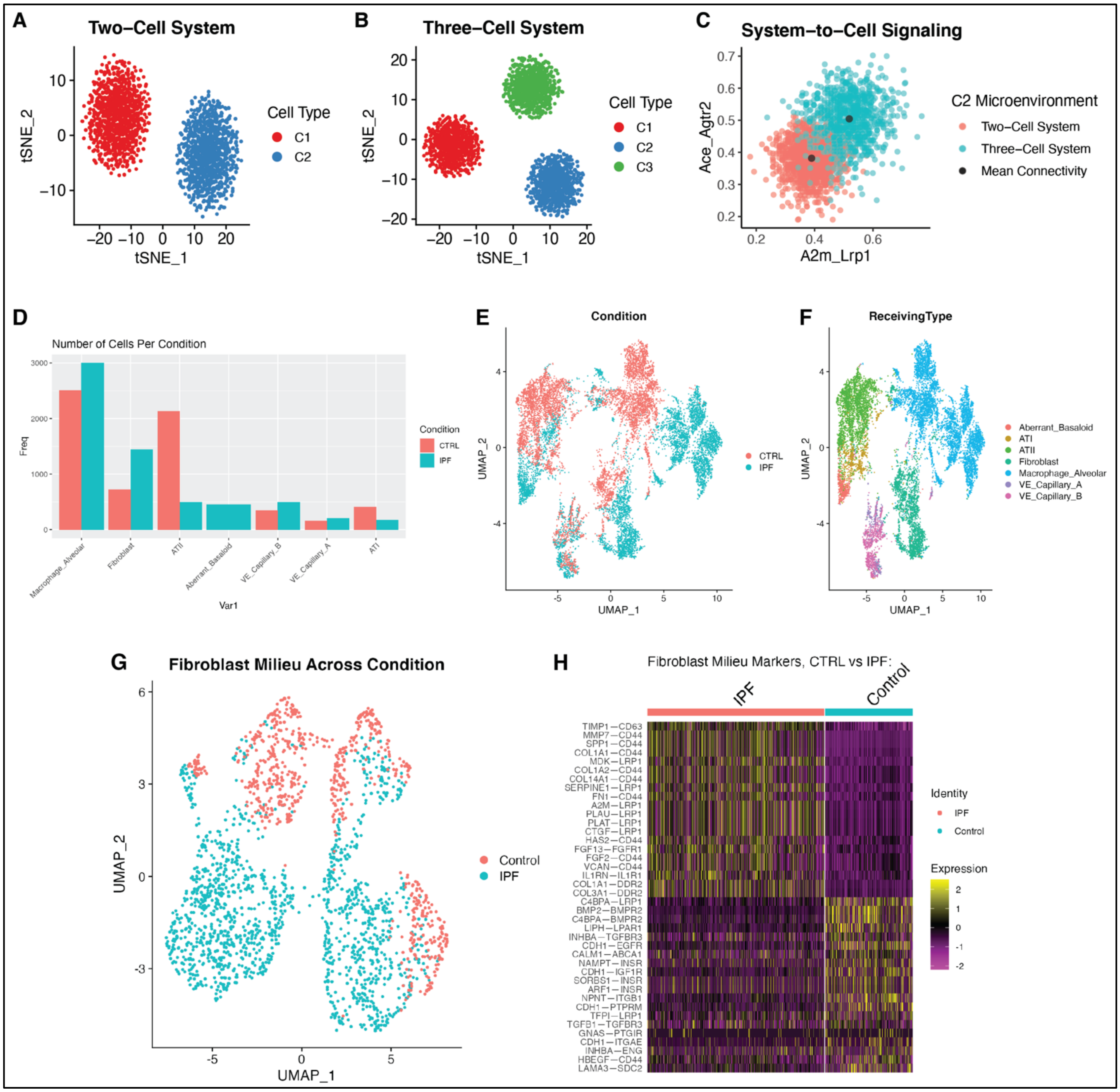

Figure S5: Differential System-to-Cell Signaling

(A-C) represent a simulation using synthetic data (see Methods), to demonstrate the capability of NICHES to measure altered system-cell signaling topology due to the addition or removal of cells. In Case 1 we have a two-cell system containing communicating cell types C1 and C2 (A). In Case 2, a third cell type (C3) has entered the system which expresses ligands cognate to receptors on C1 and C2 (B). When we use NICHES to calculate system-to-cell signaling within each case, we see a clear shift in the character of the sensed environment of celltype C2 due to the altered mean ligand expression within the system. This functionality of NICHES empowers the study of complex biological questions, such as

how added, aberrant or infiltrating cells might affect the microenvironment of a receiving celltype across experimental conditions or disease states.

(D-H) represent an applied biological example of this same use-case. In this demonstration, using data from (Adams, et al., 2019), two separate tissue systems are considered: a Control system containing 6 cell types, and an IPF condition which contains an additional aberrant cell population (annotated as "Aberrant_Basaloid") which is not identified in the control (D). We use NICHES to ask how this change in community structure influences the connectivity associated with receiving fibroblasts. (E-F) are visualizations of the System-To-Cell output from NICHES, colored by Condition and Receiving Cell Type, respectively. The measurements associated with receiving fibroblasts may be isolated (G) and differential expression testing performed to reveal mechanisms that are more- or less-prominently engaged in the fibroblast milieu across experimental condition.

Scripts to replicate the above analyses are available in the software releases associated with this publication as well as online here:
https://msraredon.github.io/NICHES/articles/09%20System%20Effects%20of%20Aberrant%20Cells.html

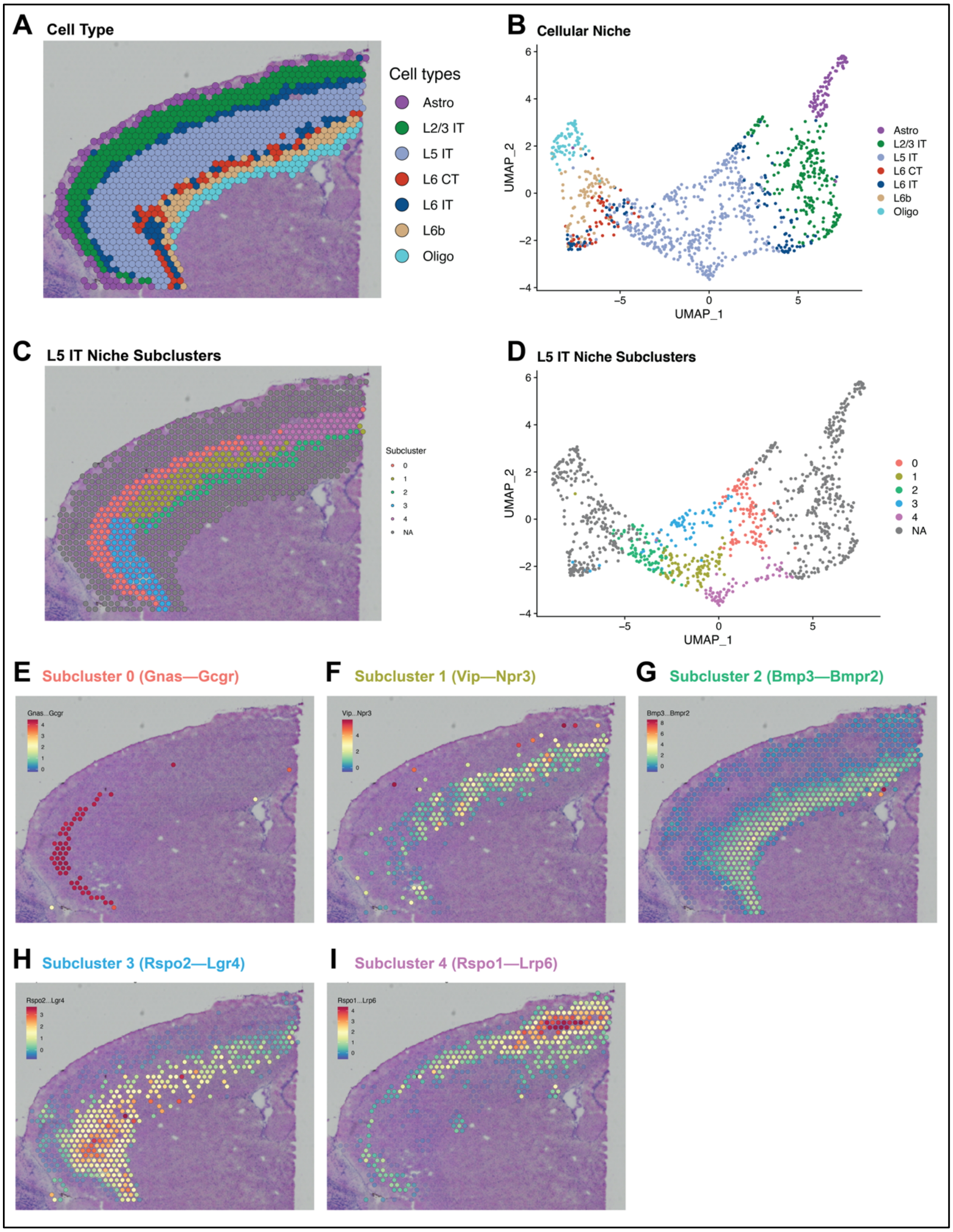

Figure S6: NICHES Reveals Intra-Celltype Microenvironment Heterogeneity

A) Spatial transcriptomic data labeled by dominant celltype (see Methods). B) UMAP embedding of cellular niche for each transcriptomic location. C) Sub-clustering of the L5 IT niche represented spatially and D) within UMAP space. Exploration of marker mechanisms reveals niche interactions specific to the microenvironments within each subcluster (E-I).

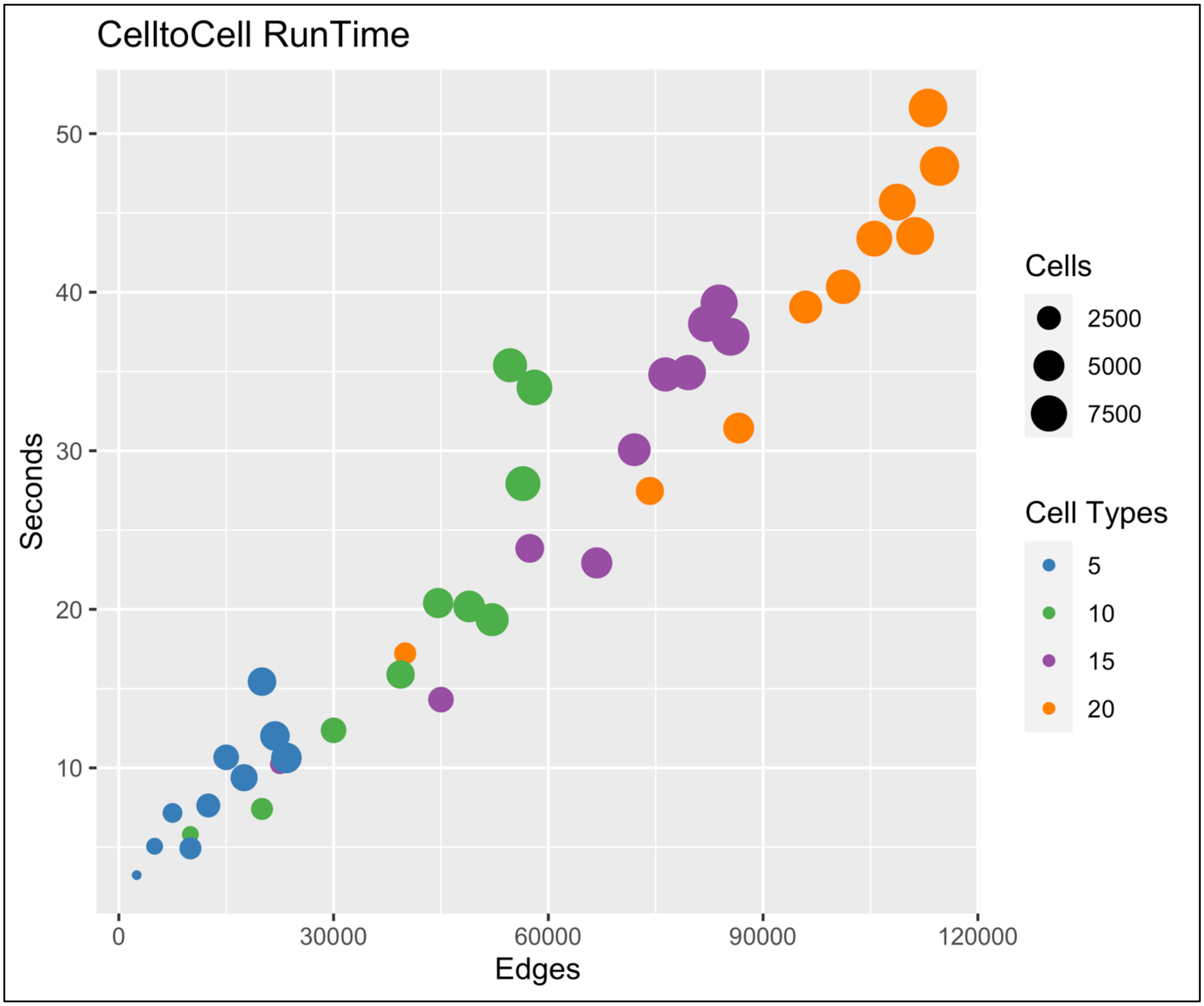

Figure S7: NICHES runtime and scalability

NICHES is designed to allow rapid analysis of cell-cell signaling patterns. Runtime scales linearly with output edge number. Edge number is a function of input cell number and cell type number and is dataset specific.

Software Methods

Dependencies

The internal workings of NICHES are dependent on a large number of other R software packages, in particular dplyr (Wickham, et al., 2019) and Seurat (Butler, et al., 2018). A maintained list of dependencies can be viewed in the DESCRIPTION file.

Mathematical and Computational Formalism

First, we define basic notations:

Table S1. Notations

Notation	Description and terminology
$\boldsymbol{C}$	$\boldsymbol{C} = \{c_1, c_2, \dots, c_{N_C}\}$. An ordered set of cells in the system where N_C is the total number of cells in the system. In the spatial transcriptomic datasets, we also use $\boldsymbol{C}$ to represent each measurement (e.g. spots).
$\boldsymbol{X}$	Normalized Gene Expression Matrix. For Cell c_i, $\boldsymbol{X}_{c_i} = \left[x_{c_i}^{g_1}, x_{c_i}^{g_2}, \dots, x_{c_i}^{g_{N_G}}\right]^T$ is its gene expression vector, where g_i is the ith gene $i \in \{1,2, \dots, N_G\}$ and where N_G is the total number of genes. For instance, $x_{c_i}^{l_k}$ is the gene expression level of ligand l_k from Cell c_i, $x_{c_j}^{r_t}$ is the gene expression level of receptor r_t from Cell c_j
$\boldsymbol{M}$	$\boldsymbol{M} = \{m_1, m_2, \dots, m_{N_M}\}$. An ordered set of ligand-receptor mechanisms where N_M is the total number of mechanisms. Each mechanism $m_k$has a corresponding ligand l_k and receptor r_k
$\boldsymbol{L}$	$\boldsymbol{L} = [l_1, l_2, \dots, l_{N_M}]$. A vector of reference ligands. Each l_k has a corresponding mechanism m_k from $\boldsymbol{M}$ in which it participates. l_k can consist of multiple subunits.
$\boldsymbol{R}$	$\boldsymbol{R} = [r_1, r_2, \dots, r_{N_M}]$. A vector of reference receptors. Each r_k has a corresponding mechanism m_k from $\boldsymbol{M}$ in which it participates. r_k can consist of multiple subunits.
$\boldsymbol{E}$	$\boldsymbol{E} \in \{0,1\}$ **,** unweighted and directed adjacency matrix that indicates which cells are connected and can interact in the system. For instance, $E_{ij} = 1$ indicates that Cell i and Cell j are connected and the ligand signals of Cell i can be received by the receptors of Cell j

Given the gene expression data $\boldsymbol{X}$ of a cell system $\boldsymbol{C}$, along with a list of known ligand-receptor mechanism $\boldsymbol{M}$, we aim to define a vector $S_{C_iC_j} \in \mathbb{R}^{N_M}$ for every connected cell pair cell i and cell j in a pre-defined cell adjacency matrix $\boldsymbol{E}$, such that $S_{C_iC_j}$ can characterize the N_M -dimensional ligand-receptor interaction profiles between cell i sending signal via ligand and cell j receiving signal via its receptors.

Cell-Cell Matrix Construction

To construct the Cell-Cell Matrix, we define $S_{C_iC_j} = \left[s_{C_iC_j}^{m_1}, s_{C_iC_j}^{m_2}, \dots, s_{C_iC_j}^{m_{N_M}}\right]^T$ in which $s_{C_iC_j}^{m_k} = x_{C_i}^{l_k} \times x_{C_j}^{r_k}$, where the mechanism m_k consists of ligand l_k and receptor r_k. We choose the multiplication operation so when the ligand or the receptor are not expressed the product is zero, representing no cell-cell signaling.

We concatenate the $S_{C_iC_j}$ Cell-Cell Interaction vectors to construct the Cell-Cell Matrix: $\boldsymbol{S} \in \mathbb{R}^{N_M \times N_E}$, where N_M is the total number of mechanisms and $N_E = \sum_{i,j}^{N_C} E_{ij}$ is the total number of (directed) connected cells.

$\boldsymbol{S}$ can be used as input to many computational analysis pipelines, including dimensionality reduction, clustering, differential expression, and trajectory inference, etc., to investigate cell-cell interactions at the individual cell level.

Computing the Adjacency Matrix $\mathbf{E}$

One step before computing a Cell-Cell Matrix is to compute the adjacency matrix $\boldsymbol{E}$. For single-cell RNA-seq datasets we assume a fully connected cellular system. However, the computational complexity of $\boldsymbol{S}$ becomes $\boldsymbol{O}(\boldsymbol{N_C^2})$, which greatly hinders the application of Cell-Cell Matrix onto cellular systems of large number of cells (e.g. $N_C > 1 \times 10^3$).

To reduce the complexity, we adopt a random sampling scheme to down-sample edges and to compute a new $\widetilde{\boldsymbol{E}}$ as follows: Let's denote the set of cell type labels in the system by $P = \{p_1, p_2, \dots, p_{N_P}\}$ where N_P is the total number of cell types. The set of cells associated with each type is denoted by $N = \{n_1, n_2, \dots, n_{N_P}\}$. For each pair of cell types within $\{(p_k, p_m) | k = 1,2, \dots, N_P; m = 1,2, \dots, N_P\}$, we draw 2 sets of cells C^{sub,p_k} and C^{sub,p_m} from cells of cell type p_k and p_m uniformly, i.e., $\{C^{sub,p_k} \subseteq C^{p_k} | \, |C^{sub,p_k}| = \min(n_k, n_m)\}$ and $\{C^{sub,p_m} \subseteq C^{p_m} | \, |C^{sub,p_m}| = \min(n_k, n_m)\}$ ($|S|$ denotes the number of elements in set S). Then we pair up C^{sub,p_k} and C^{sub,p_m}: $Q = \left\{\left(c_i^{sub,p_k}, c_i^{sub,p_m}\right) \middle| i = 1,2, \dots, \min(n_k, n_m)\right\}$. Lastly, each entry in the new adjacency matrix can be set as $\widetilde{E}_{ij} = \begin{cases} 1, & if \ (c_i, c_j) \subseteq Q \\ 0, & else \end{cases}$.

For spatial transcriptomic datasets we constrain the interactions among cells to be only within a certain distance threshold or a certain local neighborhood. To be more specific, let us denote $\boldsymbol{D}$ as the Euclidean distance matrix among cells computed from the spatial locations, where d_{ij} is the distance

between Cell i and Cell j. Given a distance threshold r, E_{ij} is computed as $E_{ij} = \begin{cases} 1, & if\ d_{ij} \leq r \\ 0, & else \end{cases}$ for each entry of $\boldsymbol{E}$ for spatial transcriptomic datasets. Alternatively, the user can specify the parameter k which computes a k-nearest neighbor (knn) graph from $\boldsymbol{D}$ and the adjacency matrix E will be computed as a mutual nearest neighbor graph from this knn graph, i.e., as $E_{ij} = \begin{cases} 1, & if\ i, j\ are\ mutual\ neighbors \\ 0, & else \end{cases}$

Niche Matrix Construction

Besides the base cell-cell interaction formulation, we extend our original definition of the Cell-Cell Matrix to investigate cellular niche and cellular influence interactions.

Specifically, we define the Niche Matrix as: $\boldsymbol{Y} \in \mathbb{R}^{N_M \times N_C}$, where N_M is the total number of mechanisms and N_C is the total number of cells in the system. A column vector of $\boldsymbol{Y}$ is defined as $Y_{C_i} = \left[y_{C_i}^{m_1}, y_{C_i}^{m_2}, \dots, y_{C_i}^{m_{N_M}}\right]^T \in \mathbb{R}^{N_M}$, i.e., each column of $\boldsymbol{Y}$ is a N_M -dimensional vector that characterizes the interaction profiles between cells sending ligand signals to Cell i which possesses the relevant receptors to receive these signals.

The connectivity value on one mechanism (e.g. m_k) between sending cells and Cell i is defined as $y_{C_i}^{m_k} = op(X^{l_k}) \times x_{C_i}^{r_k}$ where the mechanism m_k consists of ligand l_k and receptor r_k, X^{l_k} denotes the row vector of l_k's expression levels across the cells that are connected to Cell i, and $op()$ is a vector operator which, in our implementation, can be either sum (default) or $mean$.

Similarly, we define the Influence Matrix as: $\boldsymbol{Z} \in \mathbb{R}^{N_M \times N_C}$ where each column of $\boldsymbol{Z}$ is a N_M -dimensional vector that characterizes the interaction profiles between Cell i that sends the ligand signals and the cells receiving from it. Each connectivity value between Cell i and the system is defined as $z_{C_i}^{m_k} = x_{C_i}^{l_k} \times op(X^{r_k})$ where the mechanism m_k consists of ligand l_k and receptor r_k, X^{r_k} denotes the row vector of r_k's expression levels across the cells that connect to Cell i in the system, and $op()$ is again either sum (default) or $mean$.

For single-cell RNA-seq datasets without spatial coordinates, we assume a fully connected $\boldsymbol{E}$ involving all cells measured within a system. For spatial transcriptomic datasets, we construct $\boldsymbol{E}$ in the same fashion as for the spatial Cell-Cell Matrix, limiting edges to neighbors within radius r or within a user-defined set of nearest neighbors.

Metadata Mapping

NICHES allows the researcher to carry over any and all metadata (i.e. sample labels, coarse- and fine-grain cluster labeling, experimental conditions) from source data, allowing rapid downstream differential analysis between already tagged groupings of cells. For every input metadata category, the NICHES Cell-Cell Matrix output object has Sending Metadata, Receiving Metadata, and Sending-

Receiving Metadata associated with every column. The Niche Matrix and Influence Matrix have only Receiving Metadata and Sending Metadata associated with their columns, respectively.

Because each Cell-Cell Matrix contains many individual measurements of cell pairings (or environment-cell pairings in the Niche Matrix), differential analysis can be used to reveal ligand-receptor mechanisms preferential to a given celltype-celltype pairing within a system, or to identify top differential signaling mechanisms across subject, disease state, experimental condition, or tissue. Such calculations may be performed for specific celltype-celltype pairings or for other custom groupings as the user desires, based on mapped metadata. We currently recommend using ROC analysis to measure how well a mechanism differentiates two groups compared to standard two-sample tests when the columns in Cell-Cell Matrix or Niche Matrix are no longer independent.

Application Methods

Methods for Application to Synthetic Data

We generate 3 simulation datasets (*Simulation 1*, *Simulation 2*, *Simulation 3*) for 3 separate simulation analyses (Figure S3, S4, S5) respectively. For each dataset, we simulate 3 categories of genes: signaling genes (ligands and receptors), 50 non-signaling marker genes to differentiate each cell type, and 5000 noise genes. We assume the genes in the datasets follow negative binomial (NB) distributions parametrized by parameter μ which characterizes the mean expression level, and the dispersion parameter γ. We describe the exact parameter settings for each dataset as follows:

Table S2: Count matrix design for *Simulation 1*

	Cell type 1 (C1) (200 cells)		Cell type 2 (C2) (200 cells)	
	Subtype 1 (S1) (100 cells)	Subtype 2 (S2) (100 cells)	Subtype 3 (S3) (100 cells)	Subtype 4 (S4) (100 cells)
A2m	NB(μ=1, γ=20)	NB(μ=30, γ=20)	0	
Lrp1	0		NB(μ=30, γ=20)	NB(μ=30, γ=20)
Ace	NB(μ=30, γ=20)	NB(μ=30, γ=20)	0	
Agtr2	0		NB(μ=30, γ=20)	NB(μ=1, γ=20)
Marker genes (50 genes)	NB(μ=10, γ=20)		NB(μ=20, γ=20)	
Noise genes (5000 genes)	NB(μ=15, γ=20)			

Table S3: Count matrix design for *Simulation* 2 (Case 1)

	Cell type 1 (C1) (100 cells)		Cell type 2 (C2) (100 cells)
A2m	NB(μ=100, γ=20) (10 cells)	0 (90 cells)	0
Lrp1	0		NB(μ=5, γ=20)
Marker genes (50 genes)	NB(μ=10, γ=20)		NB(μ=20, γ=20)
Noise genes (5000 genes)	NB(μ=15, γ=20)		

Table S4: Count matrix design for *Simulation* 2 (Case 2)

	Cell type 1 (C1) (100 cells)	Cell type 2 (C2) (100 cells)
A2m	NB(μ=10, γ=20)	0
Lrp1	0	NB(μ=5, γ=20)
Marker genes (50 genes)	NB(μ=10, γ=20)	NB(μ=20, γ=20)
Noise genes (5000 genes)	NB(μ=15, γ=20)	

Table S5: Count matrix design for *Simulation* 3

	Cell type 1 (C1) (1000 cells)	Cell type 2 (C2) (1000 cells)	Cell type 3 (C3) (1000 cells)
A2m	NB(μ=5, γ=20)	0	NB(μ=5, γ=20)
Lrp1	0	NB(μ=30, γ=20)	0
Ace	NB(μ=5, γ=20)	0	NB(μ=5, γ=20)
Agtr2	0	NB(μ=30, γ=20)	0
Marker genes (50 genes)	NB(μ=10, γ=20)	NB(μ=20, γ=20)	NB(μ=30, γ=20)
Noise genes (5000 genes)	NB(μ=15, γ=20)		

Methods for Application to Native Lung scRNAseq Data

Data was downloaded from (Raredon, et al., 2019), subset to 4 main populations of interest, and run through standard principle component analysis (PCA), clustering, and UMAP embedding pipelines in Seurat (McInnes, et al., 2018; Stuart, et al., 2019). Data was imputed using ALRA (Linderman, et al., 2022) and then run through the NICHES function RunCellToCell. The resulting signaling matrix was then used to create a new Seurat object which was scaled and run through PCA again and embedding using UMAP. FindAllMarkers was used in Seurat to identify cell-cell interaction markers of interest.

Methods for Application to Brain Spatial Transcriptomic Data

Anterior mouse brain data was downloaded from 10x Genomics (2020) and preprocessed following the steps in Seurat (Stuart, et al., 2019), with subsetting to the frontal cortex region only. We integrated the data with a reference single-cell RNA-seq dataset (Tasic, et al., 2016) and used its cell type annotations to predict the labels of the spatial pixels by a probabilistic classifier (Seurat TransferData function). We then annotated each spatial pixel by its most probable label.

For NICHES matrix construction, we imputed the data with ALRA (Linderman, et al., 2022) based on the normalized data matrix, and then applied the NICHES Neighborhood-to-Cell function to compute niche signaling between direct histologic neighbors. The resulting niche matrix was embedded using UMAP (McInnes, et al., 2018) in Seurat 4.0 (Hao, et al., 2021). FindAllMarkers in Seurat was used to compute top markers for each celltype niche.

Supplemental References

Adams, T.S., *et al.* Single Cell RNA-seq reveals ectopic and aberrant lung resident cell populations in Idiopathic Pulmonary Fibrosis. *bioRxiv* 2019:759902.
Almet, A.A., *et al.* The landscape of cell–cell communication through single-cell transcriptomics. *Current Opinion in Systems Biology* 2021;26:12-23.
Butler, A., *et al.* Integrating single-cell transcriptomic data across different conditions, technologies, and species. *Nature biotechnology* 2018;36(5):411.
Cabello-Aguilar, S., *et al.* SingleCellSignalR: inference of intercellular networks from single-cell transcriptomics. *Nucleic Acids Research* 2020;48(10):e55-e55.
Hao, Y., *et al.* Integrated analysis of multimodal single-cell data. *Cell* 2021;184(13):3573-3587.e3529.
Hou, R., *et al.* Predicting cell-to-cell communication networks using NATMI. *Nature Communications* 2020;11(1):5011.
Jin, S., *et al.* Inference and analysis of cell-cell communication using CellChat. *Nature communications* 2021;12(1):1-20.
Linderman, G.C., *et al.* Zero-preserving imputation of single-cell RNA-seq data. *Nature Communications* 2022;13(1):192.
Lu, H., *et al.* CommPath: An R package for inference and analysis of pathway-mediated cell-cell communication chain from single-cell transcriptomics. *Computational and Structural Biotechnology Journal* 2022.
McInnes, L., Healy, J. and Melville, J. Umap: Uniform manifold approximation and projection for dimension reduction. *arXiv preprint arXiv:1802.03426* 2018.
Noël, F., *et al.* Dissection of intercellular communication using the transcriptome-based framework ICELLNET. *Nature Communications* 2021;12(1):1089.
Raredon, M.S.B., *et al.* Single-cell connectomic analysis of adult mammalian lungs. *Science Advances* 2019;5(12):eaaw3851.
Stuart, T., *et al.* Comprehensive integration of single-cell data. *Cell* 2019;177(7):1888-1902. e1821.
Tasic, B., *et al.* Adult mouse cortical cell taxonomy revealed by single cell transcriptomics. *Nature neuroscience* 2016;19(2):335.
Wang, S., *et al.* Cell lineage and communication network inference via optimization for single-cell transcriptomics. *Nucleic Acids Research* 2019;47(11):e66-e66.
Wickham, H., *et al.* Welcome to the Tidyverse. *Journal of open source software* 2019;4(43):1686.
Zhang, Y., *et al.* CellCall: integrating paired ligand–receptor and transcription factor activities for cell–cell communication. *Nucleic Acids Research* 2021.
